# Personal and professional influences on health visitors’ family focused practice for maternal mental illness: a cross sectional study

**DOI:** 10.1186/s12913-022-07499-0

**Published:** 2022-01-26

**Authors:** Rachel Leonard, Mark Linden, Anne Grant

**Affiliations:** Research Fellow, School of Nursing and Midwifery, Queen’s University, Medical Biology Centre, 97 Lisburn Road, Belfast, Northern Ireland BT9 7BL UK

**Keywords:** Maternal mental health, family focused practice, Health visiting

## Abstract

**Background:**

Family focused practice (FFP) is an approach that recognises the inter-related needs of family members and recommends a continuum of activities to support families. While it is recognised that health visitors play a key role in supporting families when mothers have mental illness, there is limited understanding of health visitor’s family focused practice (FFP) in this context and its relationships with factors, such as, workload, training, skill and knowledge, and personal and professional experience. This paper examined the effect of health visitors’ interaction with the family, and personal and professional experience on their family focused practice.

**Methods:**

A cross sectional questionnaire (Family Focused Mental Health Practice Questionnaire) was distributed to 488 health visitors within community practice in Northern Ireland, with 230 choosing to take part. Independent *t*-tests and one-way analysis of variance were used to compare family focused practice scores.

**Results:**

Results found that health visitors who had face to face contact with partners and children (t(221) = 2.61, *p* = .01), and those that directly supported the partner (t(221) = 2.61, *p* = 0.01) had a significantly higher mean score of FFP, than those that did not. However, frequency of visits (daily, weekly, monthly or yearly) had no effect on family focused practice scores. Training also had a significant effect on family focused practice scores (F(2,221) = 4.841, *p* = 0.029). Analysis of variance revealed that personal experience of mental illness had a significant effect on scores (M = 97.58, *p* = 0.009), however variables such as, age, parental status, time since registration, and being in a specialist position had no effect.

**Conclusions:**

In order for family focused practice to be effective, the quality, and content of visits and contact with family should be addressed, as opposed to a focus on the quantity of visits. However, in order for this to occur health visitors need to have appropriate support in their own right, with manageable caseloads and resources.

**Supplementary Information:**

The online version contains supplementary material available at 10.1186/s12913-022-07499-0.

## Background

Mothers’ who have mental illness and their families are recognised as prime targets for early intervention [1, 2]. Findings from systematic reviews support the need for early identification and treatment of perinatal mental illness as a potential strategy for preventing the intergenerational transmission of mental illness [3–5]. Due to the negative impact mental illness can have on the whole family, effective interventions should consider the needs of all family members through a family focused approach [6, 7]. Family focused practice (FFP) is an approach that recognises the inter-related needs of family members and recommends a continuum of activities to support families [8, 9]. Activities can range from supporting the family through supporting the mother, to addressing and supporting the shared needs of the whole family, through a whole family approach [10]. Research to date has suggested that health visitors are in a crucial position to support not only the mother’s mental health, but also the family [11]. In addition, health visitors utilise a range of family focused treatments such as the Solihull approach [12], Mellow parenting [13], and the Nurse Family Partnership [14].

Health visitors in the UK are registered nurses or midwives who have undertaken additional postgraduate training in community public health [15]. Internationally, while there are differences in how the profession is defined and referred to, there are a number of variations that are comparable. These include; health visitor in Denmark and Norway; child health nurse in Sweden; public health nurse in America, Canada, and Ireland; child and family health nurse in Australia; plunket nurse in New Zealand; social nurses in Belgium; and patronage nurse in Serbia, and Kosovo [15]. Although some countries may take different policy approaches, all professions work in the context of public health, focusing particularly on the early life of the child [16]. In the UK, health visiting is a universal service for all families that have children from 0 years to school age (4 years of age). Their core programme is centred on child health promotion, and family well-being. However, they also provide more targeted support to those families identified as having additional needs [17].

Current UK guidance advises that health visitors look beyond the child in all areas of their practice and consider the family as a whole [17]. Research that has explored the health visiting role with ‘the family’ has often mistakenly interpreted the family as the mother-infant dyad [18]. Recognising the impact of fathers’ health on the family, it is advised that fathers should also be directly involved in health visiting services, this also extends to non-resident fathers [17]. While engagement of fathers is recommended in both research and policy, it is still recognised as an area in need of improvement within health visiting [18].

While it is recognised that health visitors play a key role in supporting families when mothers have mental illness [19], there is limited understanding of health visitor’s FFP in this context and its relationships with factors such as caseload, training and personal experience of mental illness. In contrast, there is increasing understanding of FFP within other services, including adult mental health. While studies have predominately focused on barriers to FFP some have identified a number of predictors [20–22]; available services; skill and knowledge; co-worker support [21]; own parenting experience; work setting [22]; practitioner experience [22, 23]; training [23, 24]; and time and workload [22].

It is important to examine health visitors FFP and to determine factors which predict it in order to further develop practice. Within health visiting, time and workload are undoubtedly a concern due to the demands they place on the service [24, 25]. In addition, new roles within specialist perinatal and infant mental health visiting are on the increase, and have received substantial funding [19, 26]. With many believing that these specialist roles will play a valuable part in reducing the incidence and impact of maternal mental illness in the perinatal period [26, 27]. Furthermore, it has been shown that health visitors’ personal experiences can also shape their identity as a professional [28], with studies within mental health demonstrating that personal experience of mental illness can have a positive influence on understanding and establishing positive relationships with service users [28, 29].

This paper aimed to test the hypothesis that: there would be a statistically significant difference in mean FFP scores based (1) interaction with the family; (2) personal experience; (3) practice knowledge; and (4) professional experience.

## Methods

### Design

This paper reports on quantitative data from a larger study which employed a sequential mixed methods design [30]. A questionnaire was distributed by a member of the research team to all health visitors in Northern Ireland (*n* = 488) between September 2017–January 2018 with 230 choosing to take part. Ethical approval was provided by a National Health Service Research Ethics Committee (Ref 17/WS/0131). Ethical issues considered included informed consent, confidentiality and data protection.

### Participants

At time of initiation, the population consisted of 488 health visitors. A power calculation, conducted using GPower software, determined that at 80% power, with a *p* value of 0.05, and an effect size of 0.4, the required sample size was 80 participants. In order to allow for attrition, 10% was added, resulting in a final sample size of 88 health visitors. The total population of health visitors in Northern Ireland were invited to take part with 230 completing the questionnaire, indicating a response rate of 47%. Health visitors were included if they had an active caseload, had been qualified for at least 6 months and were on a permanent employment contract. Exclusion criteria consisted of those engaged in managerial roles or those who were part of the Nurse Family Partnership programme. All participants were female, however this was unsurprising given that 99% of the health visiting population in the UK are female (Department of Health, 2012). Health visitors in the sample also had a mean age of 44.31 years (SD = 9.35) (see Table 1 for participant characteristics).

**Table 1:** Characteristics of health visitors

	***n***	Family Focused Practice Score
		***Mean (SD)***
**Age Group (years)**		
25–38	74	103.45 (9.29)
39–50	77	102.54 (11.70)
51–66	72	102.32 (13.64)
Missing	0	
**Time Since Registration (years)**		
> 1–4	75	103.01 (11.04)
5–15	69	103.69 (10.06)
16–35	73	101.19 (14.07)
Missing	0	
**Service Location**		
Rural	70	101.31 (11.69)
Urban	67	102.70 (10.62)
Rural and Urban	85	102.94 (13.11)
Missing	0	
**Caseload Size**		
20–200	58	104.93 (10.75)
201–253	57	102.74 (12.08)
254–333	54	99.59 (11.31)
Missing	0	
**Mothers on Caseload with a mental illness (%)**		
1–7.5	95	101.86 (12.45)
8–15	72	104.28 (10.99)
15.50–100	17	104.94 (9.11)
Missing	0	
**Specialist health visiting position**		
Yes	52	103.54 (12.10)
No	169	102.15 (11.88)
Missing	0	
**Training: Substance misuse**		
Yes	88 (38%)	104.57 (11.71)
No	141 (62%)	101.04 (11.89)
Missing	0	
**Training: Intimate partner violence**		
Yes	91 (40%)	104.66 (11.47)
No	138 (60%)	100.90 (12.02)
Missing	0	
**Training: Perinatal mental illness**		
Yes	169 (72%)	102.50 (12.19)
No	60 (26%)	102.12 (11.18)
Missing	0	
**Training: Pre-existing mental illness (e.g. bipolar)**		
Yes	29 (13%)	104.96 (10.14)
No	200 (87%)	102.40 (12.14)
Missing	0	
**Training: Think Family Initiative**		
Yes	10 (5%)	105.00 (11.41)
No	219 (95%)	102.28 (11.95)
Missing	0	
**Training: Child focused**		
Yes	171 (75%)	102.79 (11.64)
No	58 (25%)	101.25 (12.75)
Missing	0	
**Training: Family focused**		
Yes	21 (9%)	107.95 (10.66)
No	208 (91%)	101.86 (11.92)
Missing	0	
**Experience of mental illness**		
Personal	42 (19%)	97.58 (11.41)
Family member with mental illness	85 (37%)	104.29 (10.30)
None	92 (40%)	102.70 (13.00)
Missing	10 (4%)	
**Parenting status**		
Parent	200 (87%)	102.79 (11.73)
Not a parent	29 (13%)	99.71 (13.03)
Missing	0	
**Frequency of Contact with service users**		
Daily or weekly	118 (52%)	104.76 (11.00)
Monthly or yearly	100 (43%)	100.63 (10.68)
Missing	11 (5%)	
**Discuss mental illness with female service user**		
Yes	203 (89%)	103.27 (11.10)
No	20 (8%)	97.88 (13.66)
Missing	6 (3%)	
**Contact with service users’ children**		
Yes	190 (83%)	103.34 (11.01)
No	38 (17%)	96.97 (14.53)
Missing	1	
**Contact with service users’ partner**		
Yes	186 (83%)	103.32 (11.28)
No	40 (17%)	97.78 (13.10)
Missing	0	
**Provide support to service users’ partner**		
Yes	172 (75%)	104.08 (11.29)
No	48 (21%)	97.49 (12.53)
Missing	9 (4%)	

### Measures

The questionnaire comprised three parts, demographics, the Family Focused Mental Health Practice Questionnaire (FFMHPQ) [31] and questions relating to current family focused practice.

Demographics included age, personal experience of mental illness, training and parental status. The original FFMHPQ consisted of 16 subscales expressed in 45 questions and assessed family focused behaviours in addition to organisational and professional factors which might influence family focused practice [30]. As the FFMHPQ had never before been used in a population of health visitors, the authors undertook an exploratory factor analysis which created a more parsimonious instrument consisting of 20 items, across two factors named as professional and organisational influences on family focused practice [32]. The new scale utilised a seven-point Likert scale ranging from strongly disagree – 1, to strongly agree – 7, and possessed excellent internal consistency (α = 0.949), with scores potentially ranging from 20 to 140. The third part of the questionnaire gathered data on family focused activities relating to the wider family i.e. partners and children (see supplementary data).

### Procedure

The questionnaire was distributed by a member of the research team to health visitors during staff meetings. Participants were given the opportunity to ask any questions about the study or questionnaire prior to completion. They were provided with the opportunity to either complete the questionnaire during the allotted meeting time, or could complete this in their own time, and return to the research team via a prepaid, addressed envelope.

### Data analysis

All analyses were conducted using the Statistical Package for Social Sciences 27 [33]. Independent *t*-tests were used to compare the means of binary independent variables. Variables included; face to face contact with partners; support provided to partners; face to face contact with children; discussing mental illness with mothers; and frequency of visits.

A one-way analysis of variance (ANOVA) was conducted to compare mean scores on the two-factor solution, across independent variables with more than two levels. Variables included; age; experience of mental illness; parental status; caseload size; percentage of mothers with mental illness on caseload; time since registration; being in a specialist position; and training). Post-Hoc comparisons were adjusted with Bonferroni correction to account of multiple testing. The assumptions of homogeneity were tested by the Levene’s test of variance: the assumption was met if significance was < 0.05. Results that violated this assumption are reported with the corresponding *p*-value.

## Results

### Characteristics of respondents and descriptive statistics

The questionnaire was completed by 230 health visitors from 5 Health and Social Care Trusts across Northern Ireland. Time since registration ranged from 6 months to 35 years (M = 11.39, SD = 9.54). The majority of the sample were parents (87%). The mean time in practice was 11 years (SD = 9.43), with the majority in full-time employment (*n* = 130, 57%). Caseloads ranged from 20 to 333 families. Nineteen percent (*n* = 42) of health visitors had personal experience of mental illness, 37% (*n* = 85) had experience of a family member with mental illness, and 40% (*n* = 92) had no personal or familial experience of mental illness. In relation to all the families that health visitors worked with, frequency of contact with the family comprised daily or weekly visits (56%) and monthly or yearly visits (44%). Eighty-three percent (*n* = 190) of health visitors said they had contact with children (children other than the baby), and 83% (*n* = 186) had contact with a partner. In addition, 75% (*n* = 172) of health visitors stated that they supporFted partners of mothers with mental illness. The sample had a mean FFP score of 102.40 (SD = 11.92). Scores ranged from 59 to 140. Further descriptive results are shown in Table 1.

### Differences in levels of interaction on FFP scores

Independent samples t-tests were conducted to test the hypothesis that there would be a statistically significant difference in mean FFP scores between those who had more interaction with the family than those with less.

There was a statistically significant difference in the scores for health visitors that had face to face contact with the partner (M = 103.32, SD = 11.28) compared to those that did not (*t*(221) = 2.61, *p* = .01); those with more contact had higher scores (M = 103.32; SD = 11.28) than those with less contact (M = 97.78; SD = 13.10). In addition, there was a statistically significant difference in the FFP scores for health visitors who indicated that they supported the partner (M = 104.08; SD = 11.29) compared to those who said that they did not (*t*(221) = 2.61, *p* = 0.01). There was also a significant difference in scores for those that had face to face contact with children (M = 103.34, SD = 11.01), compared to those that did not (M = 96.97; SD = 14.53) (*t*(220) = 3.00, *p* = .003). However, there was no statistically significant difference between those that discussed mental illness (M = 103.27; SD = 11.10) with the mothers compared to those that did not (M = 97.88; SD = 13.66) (*t*(215) = 1.89, *p* = .06). Through ANOVA analysis, results showed there was also a statistically significant difference of frequency of visits (daily, weekly, monthly, yearly) (F(4,211) = 8.10, *p* = .00), however post-hoc comparisons using the Bronferroni test were non-significant between all levels of frequency of contact.

### Influence of experience on FFP scores

The one-way analysis of variance was used to test the hypothesis that health visitors’ personal experience (age, experience of mental illness, and being a parent) had a significant effect on FFP.

There was a statistically significant effect of experience of mental illness (*F*(2,210) = 4.569, *p* = 0.011) on health visitors FFP. The mean scores of FFP by experience of mental illness were: personal experience, M = 97.58; No experience, M = 102.70; and having a family member with mental illness, M = 104.29. Employing the Bonferroni post-hoc test, significant differences were found between personal experience and family member experience (*p* = 0.009). There was no significant difference between personal experience and no experience (*p* = 0.1), and no experience and family member experience (*p* = 0.065). There were non-significant effects of age (*p* = 0.866) and being a parent (*p* = 0.202).

We next sought to test the hypothesis that practice knowledge and professional experience (caseload size, percentage of mums with mental illness on caseload, time since registration, being in a specialist position, and training) had a significant effect on FFP.

There was a statistically significant effect of caseload size (*F*(2,166) = 3.122, *p* = 0.047). The mean score of FFP by caseload size were: low, M = 104.93; medium, M = 102.78; and high, M = 99.60. Employing the Bonferroni post-hoc test, significant differences were found between low and high size caseloads (*p* = 0.042). There was no significant difference between low and medium size caseloads (*p* = 0.95), and medium and high size caseloads (*p* = 0.43). There was also a statistically significant effect of family focused training (F(2,221) = 4.841, *p* = 0.029), substance misuse training (*F*(1, 221) = 4.701, *p* = 0.031) and intimate partner violence (*F*(1,221) = 5.429, *p* = 0.021) on total FFMHPQ scores. There was no significant effect for perinatal mental health training (F(2,221) = 0.042, *p* = 0.837), existing mental health training (F(2,221) = 1.544, *p* = 0.215), and child focused training (F(2,221) = 0.700, *p* = 0.404). There was no significant effect of being in a specialist position; time since registration (*p* = 0.420); and percentage of mothers with mental illness on caseload (*p* = 0.328).

## Discussion

Previous research has identified variables such as; skill and knowledge [20]; personal experience of parenting [8]; work setting [21]; professional experience [21, 23]; child and family focused training [22, 23]; and time and workload [21] as having a significant effect on professionals FFP. However, many of these findings were shown to be non-significant factors in the present study. A potential explanation may lie in the variation and adaption of the FFMHPQ used in the present study compared to previous research.

Many previous studies [8, 20, 22] utilised the original FFMHPQ without psychometric evaluation and have continued to use subscales despite poor reliabilities [8, 9, 20, 21]. It was on this basis that an exploratory factor analysis was conducted for the study discussed in this paper (see [32]). Due to alterations following psychometric evaluation, the scale utilised in the present study is distinctive from both the original study and successive studies employing the FFMHPQ. This may have resulted in differing effects and relationships between the variables and FFMHPQ scores (dependent variable). Furthermore, there were some differences in how the present study and previous studies utilised the FFMHPQ in the analysis. While the present study calculated a total score of all items (*n* = 20) for the dependent variable, previous studies totalled and averaged individual subscale scores [8, 9, 20, 21]. As previously discussed, scores were not based on the same factor structures. Thus, the analysis (t-tests and ANOVA) in the present study is distinctive from previous work. Finally, previous studies have largely explored FFP within a sample of mental health professions, as opposed to health visitors, potentially explaining differing findings. Due to the differences in the FFMHPQ factor structure, approaches to the analysis, and differing populations, it may be difficult to draw comparisons from previous findings to the present study.

Despite differences with previous FFP literature, the health visiting literature nevertheless suggests that factors such as time, and workload, impact practice [34]. Results using independent t-tests found that health visitors who had face to face contact with partners and children, and those that supported the partner had a significantly higher mean score on the FFMHPQ, than those that did not. However, frequency of visits (daily, weekly, monthly or yearly) had no effect on FFMHPQ scores. This suggests that health visitors’ FFP is not dependent on workload and quantity of visits, instead it is dependent on the quality of the visit and who they have contact with.

Within the UK, health visiting caseloads are at an all-time high [34]. The Community Practitioners and Health Visitors Association (CPHVA), a UK organisation, recommend that health visitors can safely manage a maximum caseload of 250 families [34]. However, as evidenced through the present research, caseloads can be well above this limit i.e. maximum of 333. In addition to increasing caseloads, there is also greater complexity within caseloads, evidenced by the high numbers of mothers with mental illness (see Table 1). These factors have been linked to a lack of time spent with families which has been shown to be a significant barrier to effective FFP [20]. Despite high caseloads, 55% of health visitors met with families daily or weekly, and 35% visited families on a monthly basis. Indicting, that caseload size did not affect time spent with families, nor did time spent with families affect FFP. Further suggesting that effective FFP is dependent on quality and content of visits and not quantity of time spent with families.

The analyses examined whether health visitors’ professional knowledge would have a significant effect on FFMHPQ scores. In the ANOVA, training had a significant effect on FFMHPQ scores, with those who had family focused, substance misuse, and domestic violence training having significantly higher scores. Training (specifically family and child focused training) has similarly been shown to be a significant predictor of FFP in previous studies with mental health nurses [22]. However, within the sample there were varying rates of training received. For example, only 9% of the sample had received family focused training, while 75% had received child focused training. These findings call into question why so few health visitors are receiving family focused training, when it remains a policy recommendation [17].

The final hypothesis examined whether personal or professional experiences had a significant effect on FFP. ANOVA revealed that personal experience of mental illness had a significant effect on FFMHPQ scores, however variables such as, age, parental status, time since registration, and being in a specialist position had no effect on FFMHPQ scores. Health visitors’ personal experiences can influence their identity as a professional [27]. In addition, there is some evidence to suggest that personal traits such as empathy [35] sense of coherence [36], conscientiousness, and emotional stability [37] are factors which influence practice.

Among health visitors in Australia, personal experience of mental illness was associated with a deeper understanding of service users with mental illness [28, 29]. However, the present findings suggest that health visitors with personal experience of mental illness are less family focused. Many of the studies that explore professionals with personal experience of mental illness, report that professionals use this experience to guide practice with the service user [28, 38, 39]. Professionals in these studies used their experience to build rapport, trust, and relationships with the service user [38, 40, 41], viewing themselves as more authentic [42], and credible [28]. However, these studies did not explore how these experiences lead to a deeper understanding of the needs and impact beyond the service user (i.e. partner, children, grandparents), highlighting the limited existing research in this area. Thus, this study offers a unique examination of the influence of health visitors personal experience of mental illness on their understanding of the wider family. Accordingly, caution must be taken when assuming shared experience, such as mental illness, will automatically lead to better practice for all members of the family.

While the findings found that time since registration did not have a significant effect on FFP, the wider literature suggests the contrary. Indeed, there are models of nursing practice that specify that professional expertise and development is a linear process that is dependent on time [43, 44]. The more experienced (in years) the nurse, the better the quality of care for patients [45]. However, for some, these models are too limiting, and argue that the concept of expertise has been oversimplified, in that it is not solely dependent on time nor is it linear [46]. Furthermore, when stating that years in practice leads to better quality of care for service users, it is unclear who constitutes a service user. For example, in health visiting the mother and child are considered to be the primary patients/service users, while the other family members (partner) are not. It is possible that time since registration does improve quality of care for the mother or child, however, not for all family members equally. Thus, as the care of the mother and child improves, FFP decreases. However, with limited and contrary research in this area, it is difficult to determine a definitive explanation for this result.

### Methodological considerations and limitations

The FFPMHQ was a self-report tool and was thus subject to social desirability bias. While efforts were made to minimise this (e.g. participation was anonymous), the possibility of its influence exists. The sample size comprised 47% of the total available population of health visitors in NI. While this is lower than the ideal, this sample still met the underlying assumptions for our analyses and was in excess of that suggested by the power calculation. This cross-sectional study took place within the UK which is subject to country specific policies and practices. It is therefore likely that the lack of definition of FFP [47] will mean that these guiding policies will differ from those of other countries, which could therefore limit the generalisability of these findings.

## Conclusions

The evidence base for FFP is continually growing and suggests that FFP can produce positive short and long-term outcomes for all the family. In order to promote a whole family approach in health visiting, it is important to develop an understanding of their FFP and its relationship with various factors. This paper attempted to examine professional, personal and practice factors effect on health visitors’ FFP. In order for FFP to be effective, the quality, and content of visits and contact with family should be addressed, as opposed to a focus on quantity of visits. Namely, health visitors need to have contact with the child and the partner and offer appropriate support, in addition to supporting the mother, to be family focused. However, in order for this to occur health visitors need to have appropriate support in their own right, with manageable caseloads and resources. Furthermore, the findings in relation to time since registration, and personal experience, presented in this paper, are contrary to the wider literature. While shared experience, such as mental illness, and time since registration, may lead to improved practice for the mother, caution must be taken when assuming this will automatically lead to better practice for all members of the family. However, given the contradicting literature, these factors merit further investigation. Additionally, caution should be taken in drawing comparisons between the present findings and previous studies due to the differing versions of the FFMHPQ.

## Supplementary Information

**Additional file 1.**

## Data Availability

The authors confirm that the data supporting the findings of this study are available within the article and its supplementary materials.
