# Supplementary material for: Personal and professional influences on health visitors’ family focused practice for maternal mental illness: a cross sectional study
Source: BMC Health Serv Res. 2022 Jan 26;22:113. doi: 10.1186/s12913-022-07499-0 (PMC8790840; doi:10.1186/s12913-022-07499-0)
Supplement: Supplementary file 1 — Additional file 1. [file 12913_2022_7499_MOESM1_ESM.pdf]

Supplementary data for Leonard et al., (2022) Personal and professional influences on health visitors' family focused practice for maternal mental illness: a cross sectional study.

Family Focused Mental Health Practice Questionnaire Subscales

The following scale is a standardised tool designed to rate the extent to which you agree or disagree with statements regarding FFP. Items are related to FFP in relation to parents who have mental illness, their children and families. Please refer to your experience in your current position and provide a response for every item regardless of whether you have current, previous or no experience of caring for parents who have mental illness or for children whose parent(s) have mental illness. If you have no experience of caring for parents who have mental illness and/or for their children, please tick the not applicable (N/A) option for those items that ask you to comment on your actual activities with parents who have mental illness, their children and families.

In responding to the questions below, please use the following scale which ranges from (1) strongly disagree to (7) strongly agree and includes a (N/A) not applicable category. For each question, please circle the answer (number) that best corresponds with your experience.

**Not applicable**	**Strongly Disagree**	**Disagree**	**Slightly Disagree**	**Neither agree or disagree**	**Slightly Agree**	**Agree**	**Strongly agree**
**N/A**	**1**	**2**	**3**	**4**	**5**	**6**	**7**

1	My workplace provides supervision and/or mentoring to health visitors undertaking child-related work in regard to their service user-parents	**N/A**	**1**	**2**	**3**	**4**	**5**	**6**	**7**
2	There is no time to work with families or children	**N/A**	**1**	**2**	**3**	**4**	**5**	**6**	**7**
3	Government policy regarding family focused practice is very clear	**N/A**	**1**	**2**	**3**	**4**	**5**	**6**	**7**
4	Professional development regarding family focused practice is not encouraged at my work place	**N/A**	**1**	**2**	**3**	**4**	**5**	**6**	**7**
5	I often receive support from co-workers in regard to family focused practice	**N/A**	**1**	**2**	**3**	**4**	**5**	**6**	**7**
6	I regularly have family meetings (not therapy) with service users and their families	**N/A**	**1**	**2**	**3**	**4**	**5**	**6**	**7**
7	I am not confident working with service users about their parenting skills	**N/A**	**1**	**2**	**3**	**4**	**5**	**6**	**7**
8	I am able to determine the developmental progress of the children of my service users	**N/A**	**1**	**2**	**3**	**4**	**5**	**6**	**7**

Not applicable	Strongly Disagree	Disagree	Slightly Disagree	Neither agree or disagree	Slightly Agree	Agree	Strongly agree
**N/A**	**1**	**2**	**3**	**4**	**5**	**6**	**7**

9	I sometimes wish that I was better able to help service user -parents, discuss the impact of their mental illness on their children	**N/A**	**1**	**2**	**3**	**4**	**5**	**6**	**7**
10	I am knowledgeable about how parental mental illness impacts on children and families	**N/A**	**1**	**2**	**3**	**4**	**5**	**6**	**7**
11	There are no parent-related programs (e.g. parenting skills) to refer service users to	**N/A**	**1**	**2**	**3**	**4**	**5**	**6**	**7**
12	I am able to determine the level of importance that parents who have mental illness place on their children maintaining attendance at day to day activities such as school and hobbies (e.g. sport, dance)	**N/A**	**1**	**2**	**3**	**4**	**5**	**6**	**7**
13	I do not refer children of service user-parents to child focused (e.g. peer support) programs (other than child and adolescent mental health)	**N/A**	**1**	**2**	**3**	**4**	**5**	**6**	**7**
14	Working with other health and social care professionals enhances my family focused practice	**N/A**	**1**	**2**	**3**	**4**	**5**	**6**	**7**
15	My workplace does not provide supervision and/or mentoring to workers undertaking family focused practices	**N/A**	**1**	**2**	**3**	**4**	**5**	**6**	**7**
16	My workload is too high to do family focused work	**N/A**	**1**	**2**	**3**	**4**	**5**	**6**	**7**
17	At my workplace, policies and procedures for working with service user-parents on family issues are very clear	**N/A**	**1**	**2**	**3**	**4**	**5**	**6**	**7**
18	My workplace provides little support for further training in family focused practices	**N/A**	**1**	**2**	**3**	**4**	**5**	**6**	**7**
19	In my workplace other health visitors encourage family focused practice	**N/A**	**1**	**2**	**3**	**4**	**5**	**6**	**7**
20	I provide written material (e.g. education and information) about parenting to service users	**N/A**	**1**	**2**	**3**	**4**	**5**	**6**	**7**
21	I am not confident working with families of service user's	**N/A**	**1**	**2**	**3**	**4**	**5**	**6**	**7**
22	I am able to assess the level of children's involvement in their parent's symptoms	**N/A**	**1**	**2**	**3**	**4**	**5**	**6**	**7**
23	I should learn more about how to assist service user -parents about their parenting and parenting skills	**N/A**	**1**	**2**	**3**	**4**	**5**	**6**	**7**

Not applicable	Strongly Disagree	Disagree	Slightly Disagree	Neither agree or disagree	Slightly Agree	Agree	Strongly agree
N/A	1	2	3	4	5	6	7

24	I do not have the skills to work with parents about how parental mental illness impacts on children and families	**N/A**	**1**	**2**	**3**	**4**	**5**	**6**	**7**
25	There are no family therapy or family counselling services to refer service users and their families	**N/A**	**1**	**2**	**3**	**4**	**5**	**6**	**7**
26	I am able to determine the level of importance that service users place on their children maintaining strong relationships with other family members (e.g. other parent, siblings)	**N/A**	**1**	**2**	**3**	**4**	**5**	**6**	**7**
27	I refer service user to parent-related programs (e.g. parenting skills)	**N/A**	**1**	**2**	**3**	**4**	**5**	**6**	**7**
28	Children and families ultimately benefit if health professionals work together to solve the family's problems	**N/A**	**1**	**2**	**3**	**4**	**5**	**6**	**7**
29	There is time to have regular contact with other agencies regarding parents, families or children	**N/A**	**1**	**2**	**3**	**4**	**5**	**6**	**7**
30	I regularly provide information (including written materials) about mental health issues to the children of service users	**N/A**	**1**	**2**	**3**	**4**	**5**	**6**	**7**
31	I would like to undertake future training to increase my skills and knowledge for working with children of service users	**N/A**	**1**	**2**	**3**	**4**	**5**	**6**	**7**
32	I am not experienced in working with child issues associated with parental mental illness	**N/A**	**1**	**2**	**3**	**4**	**5**	**6**	**7**
33	I am not able to determine the level of importance that service users place on their children maintaining strong relationships with others outside the family (e.g. other children/peers, school)	**N/A**	**1**	**2**	**3**	**4**	**5**	**6**	**7**
34	Team-working skills are essential for all health care professionals providing family-focused care	**N/A**	**1**	**2**	**3**	**4**	**5**	**6**	**7**
35	I often consider if referral to parent support programme (or similar) is required by service users	**N/A**	**1**	**2**	**3**	**4**	**5**	**6**	**7**
36	I would like to undertake training in future to increase my skills and knowledge about helping service users with their parenting	**N/A**	**1**	**2**	**3**	**4**	**5**	**6**	**7**

Not applicable	Strongly Disagree	Disagree	Slightly Disagree	Neither agree or disagree	Slightly Agree	Agree	Strongly agree
N/A	1	2	3	4	5	6	7

37	I am skilled in working with service users in relation to maintaining the wellbeing and resilience of their children	**N/A**	**1**	**2**	**3**	**4**	**5**	**6**	**7**
38	I want to have a greater understanding of my profession in a healthcare team approach to working with children and families	**N/A**	**1**	**2**	**3**	**4**	**5**	**6**	**7**
39	I provide education sessions for adult family members (e.g. about the illness, treatment)	**N/A**	**1**	**2**	**3**	**4**	**5**	**6**	**7**
40	I am not confident working with children of service users	**N/A**	**1**	**2**	**3**	**4**	**5**	**6**	**7**
41	I am knowledgeable about the key things that parents service users could do to maintain the wellbeing (and resilience) of their children	**N/A**	**1**	**2**	**3**	**4**	**5**	**6**	**7**
